# Peimine ameliorates pulmonary fibrosis via the inhibition of M2-type macrophage polarization through the suppression of P38/Akt/STAT6 signals

**DOI:** 10.1042/BSR20220986

**Published:** 2022-10-04

**Authors:** Ze-hui Cai, Yan-ge Tian, Jun-zi Li, Peng Zhao, Jian-sheng Li, Xue Mei, Yun-ping Bai

**Affiliations:** 1 Henan Key Laboratory of Chinese Medicine for Respiratory Disease, Henan University of Chinese Medicine, Zhengzhou 450046, Henan Province, China; 2 Collaborative Innovation Center for Chinese Medicine and Respiratory Diseases co-constructed by Henan Province & Education Ministry of P.R. China, Zhengzhou 450046, Henan Province, China; 3 Zhengzhou Traditional Chinese Medicine Hospital, Zhengzhou 450006, Henan Province, China; 4 Academy of Chinese Medical Sciences, Henan University of Chinese Medicine, Zhengzhou 450000, China; 5 Department of Pathogen Immunobiology, School of Medicine, Henan University of Chinese Medicine, Zhengzhou 450000, China

**Keywords:** Akt, Macrophage, P38 MAPK, Peimine, Pulmonary fibrosis, STAT6 signal

## Abstract

Peimine, a bioactive substance isolated from Chinese medicine Fritillaria, can potentially suppress pulmonary fibrosis (PF); however, its therapeutic mechanism remains unclear. Recent evidence suggests the participation of M2**-**type macrophages in the pathogenesis of PF. The present study aimed to investigate the effect of peimine on a bleomycin (BLM)-induced PF rat model and the underlying mechanism of this effect. After BLM administration, peimine was administered to rats from day 29 to day 42, with pirfenidone (PFD) as a positive control. H&E and Masson’s trichrome stain were used to analyze histological changes. Q-PCR and western blotting were used to measure mRNA levels and protein levels, respectively. High-throughput RNA sequencing (RNA-seq) technology detected the differentially expressed genes (DEGs) regulated by peimine. Our results revealed that peimine treatment significantly ameliorated BLM-induced PF by suppressing histological changes and collagen deposition. In addition, peimine decreased the number of M2 macrophages and the expression of profibrotic factors. RNA-seq results showed that DEGs regulated by peimine in IL-4-induced macrophages were mainly associated with immune system processes, the PI3K/Akt pathway, and the MAPKs pathway. Then, immunofluorescence assay and western blot results demonstrated that peimine treatment suppressed the expression of p-p38 MAPK and p-Akt (s473) and also inhibited the nuclear translocation of p-STAT6. In conclusion, the present study demonstrated that peimine has a protective effect on PF through the suppression of M2 polarization of macrophages by inhibiting the STAT6, p38 MAPK, and Akt signals.

## Introduction

Idiopathic pulmonary fibrosis (IPF), the most common interstitial lung disease, is a major health problem whose cause is unknown [1]. IPF is characterized by the proliferation and activation of fibroblasts, the recruitment and activation of immune cells, and abnormal repair after alveolar epithelial cell injury [2]. Myofibroblasts, which are thought to be important effector cells of pulmonary fibrosis (PF), deposited a large quantity of extracellular matrix (ECM), and destroyed the normal alveolar architecture [3]. Immune cells such as alveolar macrophages are critical for the development of lung fibrosis [4], which can trigger the proliferation and activation of fibroblasts by releasing various cytokines and growth factors [5]. Macrophages are polarized to a classically activated phenotype (M1) and an alternatively activated phenotype (M2) [6]. M1 macrophages produce inflammatory factors such as tumor necrosis factor (TNF)-α, interleukin (IL)-6, and IL-12, which can promote inflammatory responses in injured tissues [7]. Following the acute phase of inflammation, M2 macrophages are induced by Th2 cytokines such as IL-4 and IL-13 [8] and produce transforming growth factor (TGF)-β, platelet-derived growth factor (PDGF), connective tissue growth factor (CTGF), and other profibrosis factors that can promote the development of fibrosis [9]. IL-4 combines with its receptor and activates the tyrosine kinase JAK, transcription factor STAT6 phosphorylation, which regulates the expression of M2 polarization markers such as arginase-1 (Arg-1) that are found in inflammatory zone 1 (Fizz-1), chitinase 3-like 3 (YM-1), and profibrotic factors [10–12]. In addition, recent studies have shown that other signal molecules such as MAPKs and Akt are also involved in this biological process [13,14].

Peimine, which is extracted from respiratory Chinese medicine Fritillaria, exerts various pharmacological properties such as anti-inflammatory, analgesic, and antioxidant properties [15,16]. For instance, peimine can decrease the levels of inflammatory cytokines produced by LPS-induced macrophages through the suppression of the MAPKs and NF-KB signaling pathways and inhibit cigarette smoke-induced oxidative stress in macrophages [17,18]. However, its potential role in treating PF is yet to be understood.

We hypothesized that peimine suppresses M2-type macrophages by inhibiting the STAT6 transcription factor, p38 MAPK, and Akt, and could be a new strategy for relieving PF. We used the bleomycin (BLM)-induced PF rat model and the IL-4-induced M2 polarization cell model to investigate the antifibrosis and M2 polarization effects of peimine. High-throughput RNA sequencing (RNA-seq) technology was used to detect the differentially expressed genes (DEGs) regulated by peimine in M2 macrophages. The critical pathways regulated by these genes were validated in peimine-treated M2 macrophages.

## Materials and methods

### Materials and reagents

Peimine (CAS: 23496-41-5) was prepared by Chengdu Munster Biotechnology Co., Ltd. (Chengdu, Sichuan, China). Bleomycin sulfate was obtained from Selleck (lot S1214) (Shanghai, China). Pirfenidone (PFD) capsules were obtained from the Beijing Kangdini Pharmaceutical Co. Ltd. (lot 150603) (Beijing, China). Antibodies against Collagen I, Collagen III, and CD68 were purchased from Affinity (AF7001, AF0136, DF7518), while antibodies against Arg-1, CD206, CTGF, TGF-β1, and glyceraldehyde-3-phosphate dehydrogenase (GAPDH) were obtained from Proteintech (16001-1-AP, 60143-1-Ig, 23936-1-AP, 21898-1-AP, 60004-1-Ig). P-STAT6, STAT6, p-Akt (s473), Akt, p-p38 MAPK, p38 MAPK antibodies were obtained from Cell Signaling Technology (5397S, 56554S, 4060S, 4685S, 4511S, 8690S). FITC and Cy3-conjugated affinipure goat antirabbit IgG (H+L) were purchased from Proteintech (SA00003-2, SA00009-2).

### Animals and cells

Sprague–Dawley rats (half male and half female, 6–8 weeks old, weight 200 ± 10 g) were provided by Jinan Pengyue Experimental Animal Breeding Co., Ltd. (Jinan, Shandong, China). The rats were fed adaptively for 1 week in the Animal Experiment Center of Henan University of Traditional Chinese Medicine, a well-ventilated environment with a temperature of 24 ± 2°C, relative humidity of about 50%, light (12-h light/12-h dark cycle), and fed with pure water and aseptic feed (SPF Biotechnology Co., Ltd., Beijing, China). This experiment was reviewed and approved by the Experimental Animal Care and Ethics Committee of the First Affiliated Hospital of Henan University of Traditional Chinese Medicine (Batch number: DWLL202003210).

The mouse alveolar macrophages MH-S cells (obtained from Cell Resource Center of Shanghai Academy of Life Sciences, Chinese Academy of Sciences, Shanghai, China) were cultured in RPMI 1640 (Beijing Soleibao Science & Technology Co., Ltd., Beijing, China) containing 10% FBS (Lonsrea, Uruguay) and maintained at 37°C and 5% CO_2_ in an incubator for static culture.

### Model preparation and administration

Forty-eight rats were randomly divided into four groups (the normal, model, peimine, and PFD groups) after being fed adaptively for 1 week. There were 12 (six females and six males) rats in each group. After anesthetizing the rats with 3% pentobarbital sodium (2.5 ml/kg), those in the model, peimine, and PFD groups were induced by a one-time intratracheal administration of bleomycin dissolved in normal saline (5 mg/kg) to establish the rat model of PF [19]. The rats in the normal group were administered the same volume of normal saline. The rats were anesthetized with 3% pentobarbital sodium (2.5 ml/kg), and endotracheal intubation was performed to assess the pulmonary function. Then, the rats were sacrificed by blood sampling through the abdominal aorta.

### Gene function analysis

Gene ontology (GO) enrichment analyses (*P-*value<0.05) of DEGs in the IL-4 induced MH-S model group and the peimine group were performed to investigate functions from the three aspects (biological processes, cellular components, and molecular functions). The Kyoto Encyclopedia of Genes and Genomes (KEGG) analysis (*P-*value<0.05) was used to classify the biological pathway clusters covering the different genes in the IL-4-induced MH-S model group and the peimine group.

### Histomorphology and immunohistochemical analysis

Left lung tissues of rats were perfused with 10% neutral paraformaldehyde and fixed for 72 h at room temperature, and the formaldehyde fixation solution was changed every 24 h thereafter. With lung hilum as the center, pulmonary tissue blocks with a thickness of about 3 mm were continuously cut along the short diameter for paraffin embedding. Then, the embedded wax blocks were cut into 4-μm-thick slices and stained with H&E and Masson’s trichrome. The severity of pulmonary interstitial fibrosis in rats was evaluated using the Ashcroft scoring system [20]. For immunohistochemical analyses, lung tissue sections were stained with the first antibody (Collagen I, Collagen III, CD68, Arg-1, CD206, CTGF, TGF-β1), followed by staining with the secondary antibody of the corresponding species. The contents of absolute collagen and other protein indexes were assessed via semiquantification by integrated optical density (IOD) using the Image-Pro Plus 6.0 image analysis software program (Media Cybernetics, USA).

### Quantitative real-time PCR analysis

MH-S cells (1 × 10^6^) were challenged with IL-4 (20 ng/ml) for 6 h, followed by incubation with peimine (0, 12.5, 25, 50, and 100 μg/ml) and 0.1% dimethyl sulfoxide (DMSO) as usual for 3 h. The total RNA of cells was extracted using the QIAzol Lysis Reagent (Qiagen, USA). cDNA was synthesized using the HiScript® II 1st Strand cDNA Synthesis Kit (Vazyme, Nanjing, China) and amplified by ChamQ™ Universal SYBR® qPCR Master Mix (Vazyme, Nanjing, China). The primer sequences are shown in Table 1. The relative mRNA expression of target genes was obtained via the 2^−△△CT^ method and normalized to the housekeeping gene *GAPDH*.

**Table 1:** The primers used in cellular experiments

Gene	Primer 5′→3′
Arg-1	Forward-TGTCCCTAATGACAGCTCCTT
	Reverse-GCATCCACCCAAATGACACAT
Fizz-1	Forward-CCAATCCAGCTAACTATCCCTCC
	Reverse-ACCCAGTAGCAGTCATCCCA
TGF-β1	Forward-CTCCCGTGGCTTCTAGTGC
	Reverse-GCCTTAGTTTGGACAGGATCTG
CTGF	Forward-GACCCAACTATGATGCGAGCC
	Reverse-CCCATCCCACAGGTCTTAGAAC
GAPDH	Forward-AGGTCGGTGTGAACGGATTTG
	Reverse-GGGGTCGTTGATGGCAACA

### Western blotting analysis

MH-S cells were plated in 35-mm cell culture dishes at 1 × 10^6^/dish overnight and then cells were treated with peimine (0, 12.5, 25, 50, and 100 μg/ml) and 0.1% DMSO for 3 h before being challenged with IL-4 (20 ng/ml) for 6 h. Cultured cells were collected and lysed in 1 × RIPA buffer (EMD Millipore, USA) plus pierce protease and phosphatase inhibitor minitablets (Thermo Scientific, USA). The protein concentrations were determined using the BCA protein assay kit (Solarbio, Beijing, China). For CD206, Arg-1, p-STAT6, STAT6, p-p38 MAPK, p38 MAPK, p-Akt (s473), Akt, and GAPDH, 20 μg of total cellular proteins was separated by 10% SDS-PAGE and then transferred to 0.2-µm immobilon polyvinylidene difluoride filter (PVDF) membranes (Millipore, Ireland). After being blocked with 5% skim milk at room temperature for 1 h, the membranes were incubated with specific primary antibodies at 4°C overnight. The next day, after at room temperature for 30 min, the membranes were incubated with an HRP-conjugated secondary antibody at 37°C for 1 h. The protein bands were detected using an ECL Western Blotting Substrate (Affinity, USA) and the images were captured using the ChemiDoc MP imaging system (Bio Rad, USA).

### Immunofluorescence assay

MH-S cells were grown in 35-mm confocal dishes (BioSharp, China). After incubating with IL-4 (20 ng/ml) for 6 h and the removal of the culture medium, the cells were washed twice with PBS for 5 min, fixed in 4% paraformaldehyde at room temperature for 15 min, washed again twice with PBS for 5 min, then permeabilized with 0.3% Triton X-100 in PBS for 30 min. After being washed twice with PBS for 5 min, the cells were blocked with 10% goat serum at room temperature for 2 h and incubated with primary antibody for CTFG (1:200) or TGF-β1 (1:200) overnight at 4°C. The next day, after being kept at room temperature for 1 h, the cells were washed three times with PBS and then with an appropriate fluorophore-conjugated secondary antibody for 1 h at room temperature in the dark. After being washed twice with PBS and once with ddH_2_O, DAPI (100 nM) was used to stain the cells’ nuclei for 2 min in the dark. Finally, after being washed once with ddH_2_O, the cells were kept in a mounting medium before images were captured [21] using a laser scanning confocal microscope (Carl Zeiss LSM700, Germany).

### Statistical analysis

Statistical analyses were carried out using IBM SPSS Statistics 22.0 (SPSS, Inc., Chicago, IL, USA). The normal, model, and different drug groups were compared using the one-way ANOVA test. The least significant difference (LSD) was used for those with a normal distribution and uniform variance while Dunnett’s T3 was used for those with a normal distribution but uneven variance. The data were presented as the mean ± standard error of the mean (SEM). *P*<0.05 was considered statistically significant.

## Results

### Peimine-alleviated BLM-induced PF

**Figure 1:**
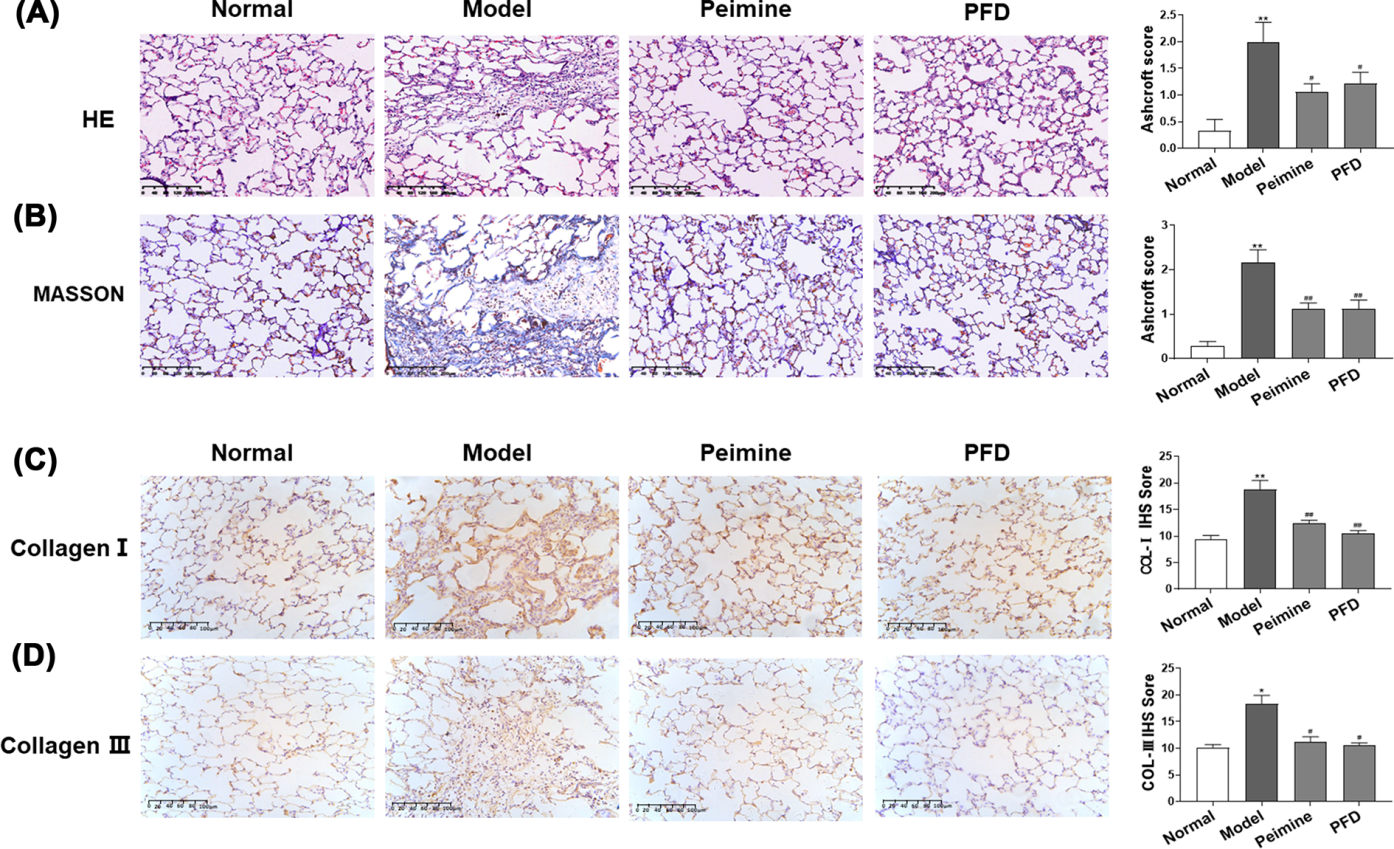
Effects of peimine on BLM-induced PF in rats (**A,B**) H&E staining and Masson staining of the pulmonary tissue. Left panel: Representative results for H&E and Masson. Right panel: Bar graphs showing the Ashcroft scores. (**C,D**) Immunohistochemical staining for COL I and COL III in pulmonary tissue. Left panel: Representative results for COL I and COL III. Right panel: Bar graphs showing the IOD scores. Scale bar: 200 μm. Data were expressed as means ± SEM (*n*=6). *P<0.05 and **P<0.01 vs. the normal group; ^#^*P*<0.05 and ^##^*P*<0.01 vs. the model group.

To detect its therapeutic effect, peimine (0.24 mg/kg) was orally administered to rats from day 29 to day 42 after the instillation of BLM. As shown in the results of H&E and Masson’s trichrome staining, the lungs of PF-affected rats showed collapsed alveolar structure destruction, inflammatory cell infiltration, fibroblast proliferation, and excessive collagen deposition. In addition, these changes were significantly suppressed by peimine treatment. (Figure 1A,B; *P*<0.05, *P*<0.01). Consistently, peimine-treated rats showed a decrease in the expression of the collagen I (COL I) and collagen III (COL III) (Figure 1C,D; *P*<0.05, *P*<0.01). In addition, PFD displayed a similar ameliorative effect on histological changes and collagen deposition. Taken together, peimine showed a significant anti-PF effect with a potency close to that of PFD.

### Peimine suppressed the M2 polarization of macrophages

**Figure 2:**
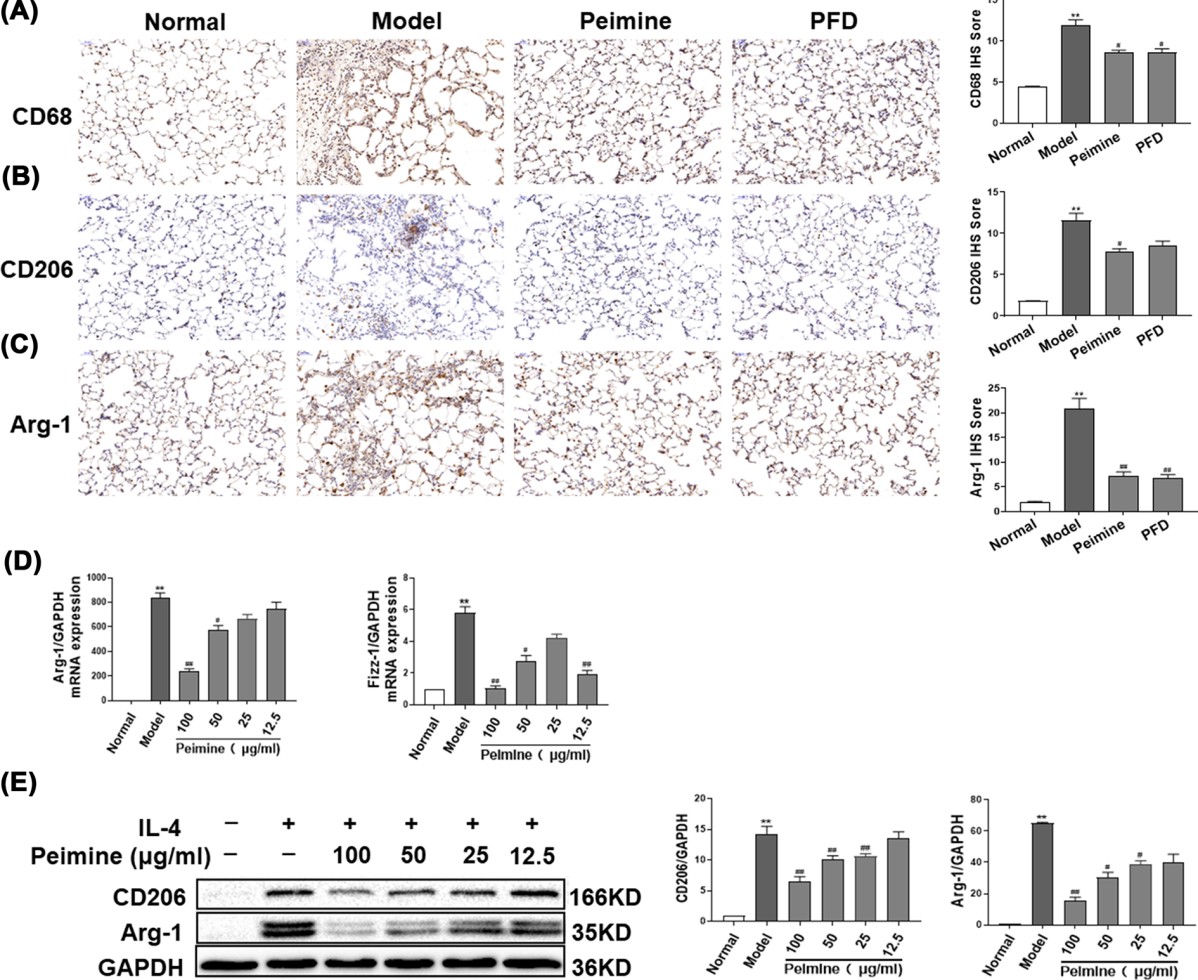
Peimine attenuates M2-type polarization of macrophages on BLM-induced PF in rats and IL-4-induced MH-S cells (**A–C**) Immunohistochemical staining for CD68, CD206, and Arg-1 in pulmonary tissue. Left panel: Representative results for CD68, CD206, and Arg-1. Right panel: Bar graphs showing the IOD scores. Scale bar: 200 μm. Data were expressed as means ± SEM (*n*=6). **P<0.01 vs. the normal group; ^#^*P*<0.05 and ^##^*P*<0.01 vs. the model group. (**D**) The expression of Arg-1, Fizz-1 in MH-S cells was evaluated by quantitative PCR. (**E**) The expression of Arg-1, CD206 in MH-S cells was evaluated by western blot. Left panel: Representative western blot results. Right panel: Bar graphs showing the mean data in each group. Data were expressed as means ± SEM (*n*=3). **P<0.01 vs. the normal group; ^#^*P*<0.05 and ^##^*P*<0.01 vs. the model group.

Macrophages can generate proinflammatory cytokines and profibrotic mediators that lead to inflammatory cell infiltration and myofibroblasts activation. We detected the expression of the macrophage marker, CD 68, and the M2 markers, CD206 and Arg-1, in lung tissues. Immunohistochemical results indicated that the expression of CD68, CD206, Arg-1 in BLM-induced rats was substantially increased and significantly inhibited by peimine treatment (Figure 2A–C; *P*<0.05, *P*<0.01). To investigate the effect of peimine on M2 polarization, peimine was exposed to alveolar macrophages induced by IL-4. As shown in Figure 2D, a high concentration of peimine can significantly reduce the mRNA expression of Arg-1 and Fizz-1 in macrophages induced by IL-4 (Figure 2D; *P*<0.05, *P*<0.01). In addition, we found that peimine decreased the protein level of Arg-1 and CD206 induced by IL-4 (Figure 2E; *P*<0.05, *P*<0.01). Taken together, these results indicated that peimine can reduce the total number of macrophages and M2 macrophages in PF-affected lung tissues.

### Peimine decreased the expression of fibrotic factors *in vivo* and *in vitro*

**Figure 3:**
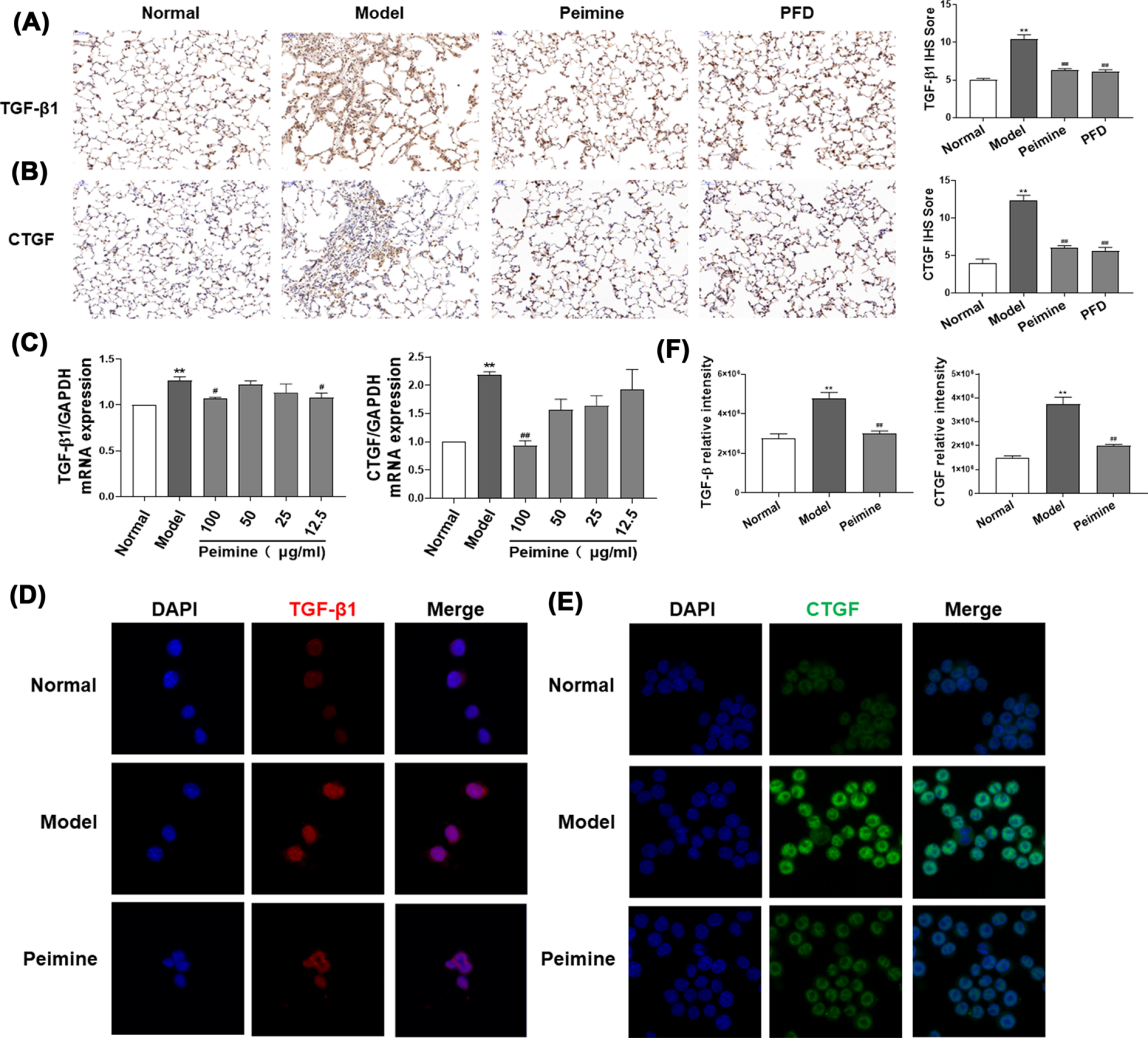
Peimine relieves the expression of fibrotic factors on BLM-induced PF in rats and IL-4-induced MH-S cells (**A,B**) Immunohistochemical staining for TGF-β1 and CTGF in pulmonary tissue. Left panel: Representative results for TGF-β1 and CTGF. Right panel: Bar graphs showing the IOD scores. Scale bar: 200 μm. Data were expressed as means ± SEM (*n*=6). **P<0.01 vs. the normal group; ^##^*P*<0.01 vs. the model group. (**C**) The expression of TGF-β1 and CTGF in MH-S cells was evaluated by quantitative PCR. Data were expressed as means ± SEM (*n*=3). **P<0.01 vs. the normal group; ^#^*P*<0.05 and ^##^*P*<0.01 vs. the model group. (**D–F**) The expression of TGF-β1 and CTGF in MH-S cells was evaluated by immunofluorescence staining. (**D,E**) Immunofluorescence staining results (400×). (**F**) Bar graphs showing the mean data in each group. Data were expressed as means ± SEM (*n*=9). **P<0.01 vs. the normal group; ^##^*P*<0.01 vs. the model group.

M2 macrophages generate various profibrotic factors, such as TGF-β1, CTGF, and PDGF, which induce the development of PF [5]. Thus, we detected the level of profibrotic factors in lung tissues and M2 macrophages, and observed an increase in the expression of CTGF and TGF-β1 in BLM-induced rat lung tissues; however, peimine could inhibit this increase (Figure 3A,B; *P*<0.05, *P*<0.01). To determine whether peimine treatment could decrease the expression of profibrotic factors in M2 macrophages, we measured the mRNA expression of CTGF and TGF-β1 in IL-4-induced macrophages. The result suggested that high concentrations of peimine can reduce the mRNA expression of fibrotic factors (Figure 3C; *P*<0.05, *P*<0.01). Immunofluorescence staining further demonstrated that peimine can reduce the protein levels of CTGF and TGF-β1 in M2 macrophages (Figure 3D–F). Taken together, these results indicated that peimine can reduce the various profibrotic factors generated by M2 macrophages.

### Functional prediction of aberrantly expressed genes

**Figure 4:**
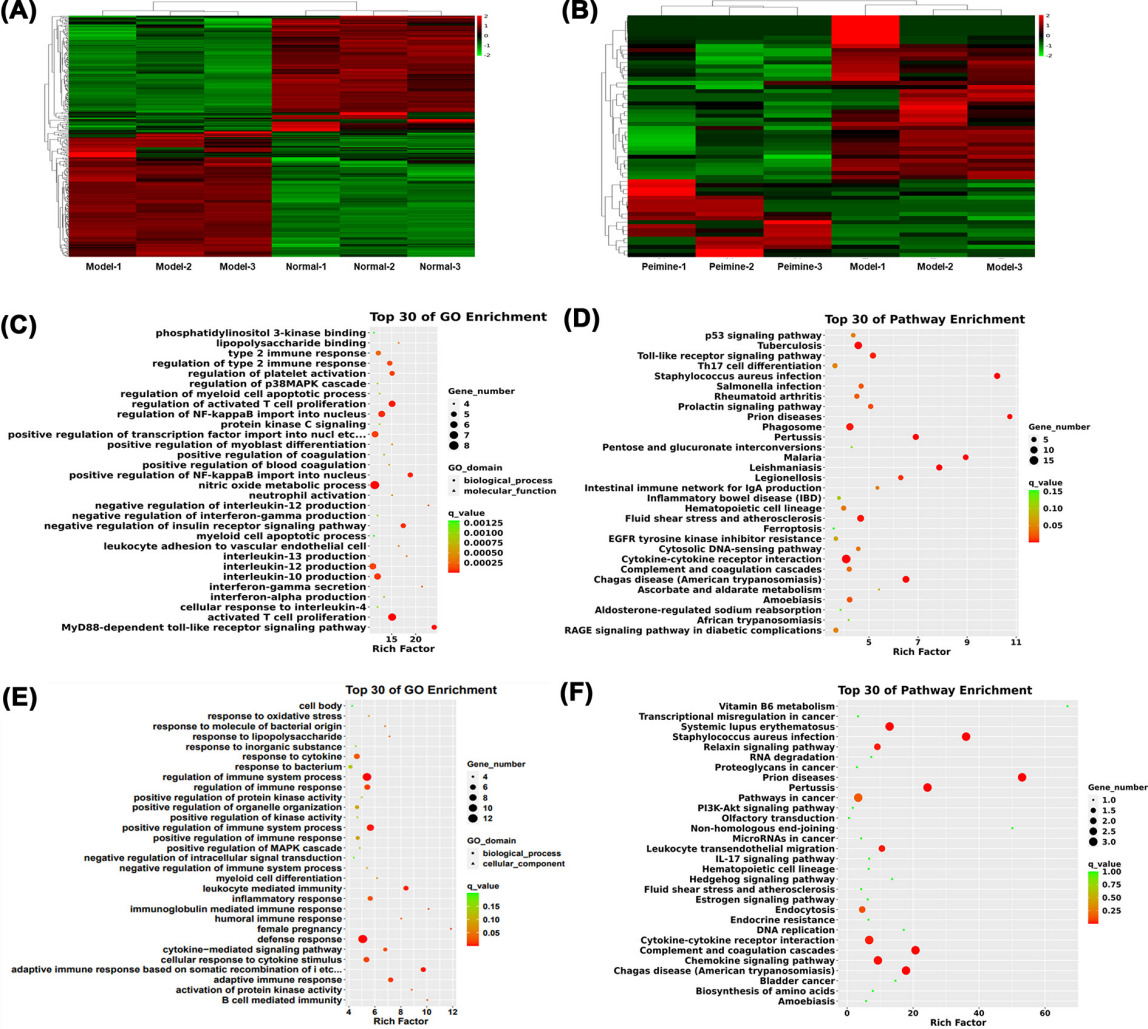
The identification of DEGs (**A,B**) The heat-map showed the hierarchical clustering of differential expression in mRNAs. Left panel: The model group vs. the normal group. Right panel: The peimine group vs. the model group. (*P*<0.05, |log2FC| > 1). Up-regulated genes were indicated as ‘red,’ and down-regulated genes were indicated as ‘green,’ *n*=3 for each group. (**C,D**) Top of 30 GO and KEGG pathway enrichment, the model group vs. the normal group. (**E,F**) Top of 30 GO and KEGG pathway enrichment, the peimine group vs. the model group.

We used high-throughput sequencing to assess the DEGs regulated by peimine treatment. As a result, a total of 298 DEGs (|log2FC| ≥ 1, *P-value*<0.05), including 155 up-regulated and 143 down-regulated genes, were identified between the model group and the normal group. Nineteen up-regulated and 40 down-regulated genes were determined between the model group and the peimine group (|log2FC| ≥1, *P-value*<0.05). Using hierarchical clustering, the DEGs displayed two significant variations in heat maps (Figure 4A,B). To further clarify the potential biological functions of these DEGs, we performed the GO enrichment and KEGG pathway enrichment analyses. The results indicated that DEGs in IL-4-treated macrophages (vs. normal macrophages) were associated with various biological processes, such as PI3K binding and the regulation of the p38 MAPK cascade (Figure 4C), and multipathways, including the p53 signal pathway, Toll-like signals, and Th17 cell differentiation (Figure 4D). In peimine-treated macrophages (vs. IL-4-treated macrophages), the results indicated that DEGs were also associated with various biological processes such as the regulation of immune system process, defense responses, the positive regulation of immune system processes (Figure 4E), and multiple pathways, including the PI3K/Akt signaling pathway (Figure 4F). In summary, peimine may regulate the immune system, the PI3K/Akt pathway, and the MAPKs pathway.

### Peimine relieves IL-4-induced M2 polarization via the regulation of the STAT6 transcription factor, the p38 MAPK pathway, and the Akt pathway

**Figure 5:**
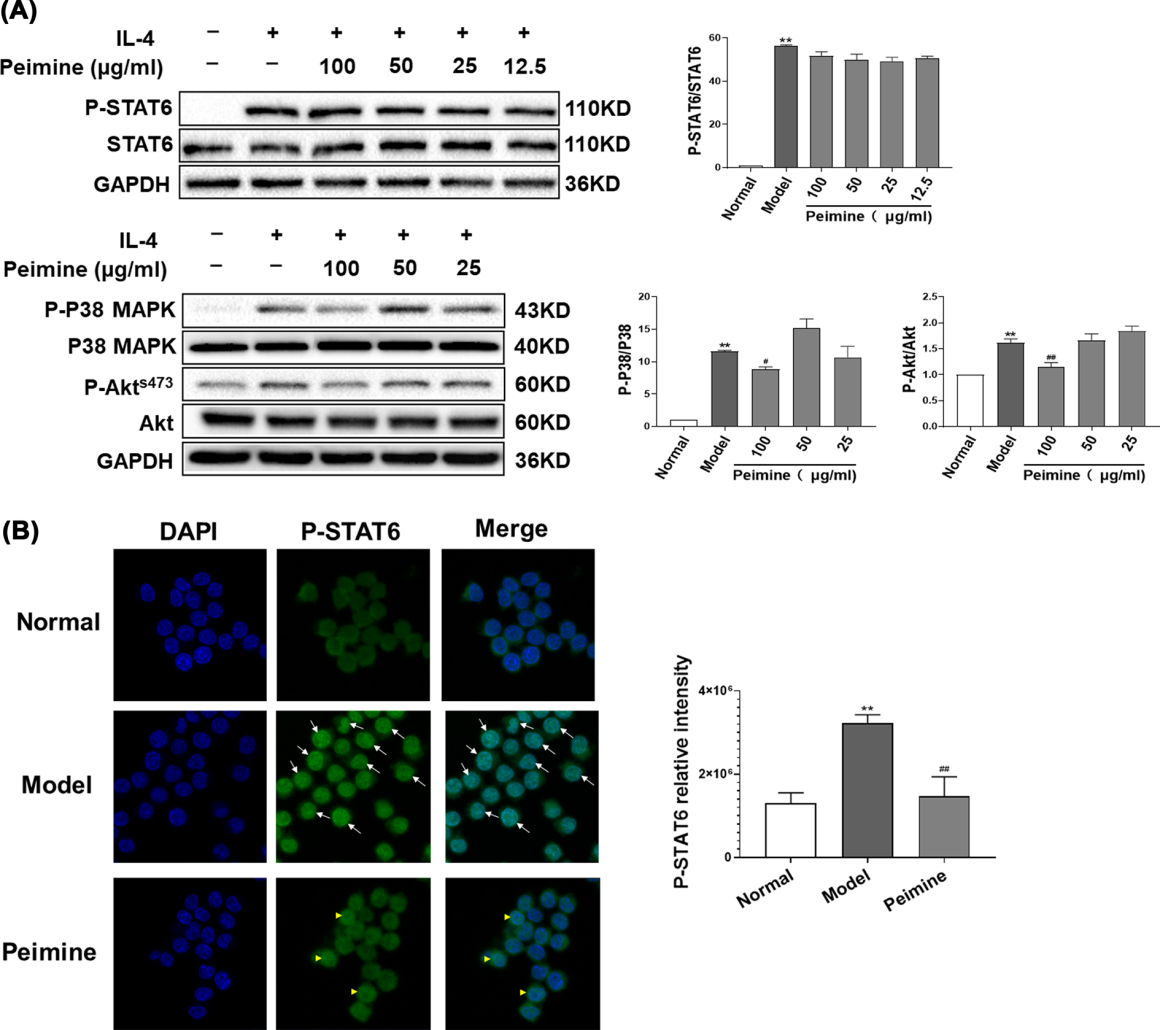
Peimine relieves IL-4-induced M2 polarization via regulation of the STAT6 transcription factor, P38 MAPK, and Akt (**A**) The expression of p-STAT6, STAT6, p-p38 MAPK, p38 MAPK, p-Akt (s473), Akt in MH-S cells was evaluated by western blot. Left panel: Representative western blot results. Right panel: Bar graphs showing the mean data in each group. Data were expressed as means ± SEM (*n*=3). **P<0.01 vs. the normal group; ^#^*P*<0.05 and ^##^*P*<0.01 vs. the model group. (**B**) The expression of p-STAT6 in MH-S cells was evaluated by immunofluorescence staining. Left panel: Immunofluorescence staining results (400×). Right panel: Bar graphs showing the mean data in each group. Data were expressed as means ± SEM (*n*=9). **P<0.01 vs. the normal group; ^##^*P*<0.01 vs. the model group.

To further explore the mechanisms of antipolarization of M2 macrophages of peimine, we detected the protein levels of p-STAT6, p-p38 MAPK, and p-Akt (s473), three important pathways for the induction of M2 differentiation [22]. As shown in Figure 5, peimine treatment could decrease the protein levels of p-p38 MAPK and p-Akt (s473) (*P*<0.01) but had no effect on the protein expression of p-STAT6 (Figure 5A), However, peimine could inhibit the nuclear translocation of p-STAT6 (Figure 5B). These data suggest that peimine could inhibit the p38 MAPK, Akt, and STAT6 signals involved in the IL-4-induced M2 polarization of macrophages.

## Discussion

IPF is characterized by fibroblast activation and collagen deposition [23,24]. It has been estimated that the socioeconomic burden of IPF is substantial with the aging population worldwide [25]. At present, the evidence-based treatment guidelines of IPF recommend the use of pirfenidone and nintedanib [26–28]. However, the efficacy of these two drugs in preventing disease progression and improving patients’ quality of life is limited [29,30]. Therefore, there is an urgent need to search for an effective clinical treatment for IPF. Peimine, an alkaloid extracted from the traditional Chinese medicine Fritillaria, has been demonstrated to have anti-inflammatory, analgesic, and antioxidant properties [15,16]. In the present study, the potential beneficial effects of peimine were investigated in BLM**-**induced PF-affected rats. The BLM-induced PF model, which shows similar characteristics to IPF, is widely used in fibrosis investigations [31]. In the present work, we treated BLM-induced PF-affected rats with peimine on day 29 and investigated whether peimine has a beneficial effect in the ‘fibrotic’ phase of fibrosis development. The results demonstrated that peimine significantly improved the pathological damage of lung tissues and reduced the expression of COL I and COL III in PF-affected rats.

Macrophages mainly comprise M1 and M2 populations. M1 macrophages, which play a critical role in the onset of lung fibrosis, typically participate in inflammation, whereas M2 macrophages are associated with tissue repair and remodeling, which is the progression of fibrosis. Accumulating evidence has shown that the M2 polarization of macrophages contributes to the development and progression of IPF through the secretion of cytokines [32] such as TGF-β1 and CTGF [5,33,34]. For instance, pirfenidone exerts its antifibrotic property in part by suppressing the TGF-β expression relevant to macrophage M2 polarization and fibroblast activation [35]. Furthermore, Neotuberostemonine (NTS), one of the traditional Chinese medicines included in all versions of the Chinese Pharmacopoeia, can effectively attenuate BLM-induced PF by suppressing the recruitment and polarization of M2 macrophages [36]. However, there is still a pressing need for new therapeutic approaches as current therapies are unable to effectively attenuate disease progression and reverse lung fibrosis. Here, we found that peimine could decrease the number of M2 macrophages in lung tissues, inhibit M2 polarization, and profibrotic cytokine secretion in alveolar macrophages induced by IL-4. It implied that peimine could ameliorate PF probably by inhibiting the M2 polarization of macrophages.

RNA-Seq analysis showed that the DEGs in IL-4 and/or peimine-treated macrophages were mostly related to the immune system process, the PI3K/Akt pathway, and the MAPKs pathway. The MAPKs signal pathway participates in multiple cellular activities and responds to different kinds of cellular stress [37]. A previous study demonstrated that IL-4 could up-regulate the phosphorylation of p38 MAPK and induce M2 polarization in macrophages, and p38 inhibitors could diminish the level of M2 makers in macrophages induced by IL-4 [22]. Akt signal activation is required for M2 polarization [38]. IL-4 treatment results in the parallel and independent activation of Akt/mTORC1 and the canonical JAK/STAT signals, which both drive the transcription of STAT6 responsive genes such as Arg-1 [39]. The inhibition of p38 MAPK can significantly inhibit Akt and STAT6 phosphorylation, suggesting that p38 MAPK is upstream of these pathways [22]. In the present study, we found that peimine treatment could suppress p-p38 MAPK and p-Akt (s473) expression in IL-4-induced macrophages and that peimine had no effect on the expression of p-STAT6 but inhibited the nuclear translocation of p-STAT6.

In summary, we demonstrated that peimine has a protective effect on BLM-induced PF-affected rats through the suppression of the M2 polarization of macrophages by inhibiting p38 MAPK/Akt/STAT6 signals. These findings suggest that peimine may be a viable drug for the treatment of PF. However, many questions remain unanswered regarding the exact mechanisms of the manipulation of the balance of M1/M2 phenotypes in the pathogenesis of IPF. Future studies aimed at dissecting the interplay between macrophages and fibroblasts and investigating how macrophages create an extracellular milieu that favors fibroblast activation and myofibroblast proliferation would be crucial for developing effective therapies against IPF and other fibrotic diseases.

## Data Availability

The data associated with the paper can be accessed from the first author.

## Abbreviations

Arg-1: arginase-1
BLM: bleomycin
COL I: collagen I
CTGF: connective tissue growth factor
DEG: differentially expressed gene
DMSO: dimethyl sulfoxide
ECM: extracellular matrix
FBS: Fetal Bovine Serum
GAPDH: glyceraldehyde-3-phosphate dehydrogenase
GO: gene ontology
IL: interleukin
IOD: integrated optical density
IPF: Idiopathic pulmonary fibrosis
KEGG: Kyoto Encyclopedia of Genes and Genomes
LSD: least significant difference
NTS: Neotuberostemonine
PDGF: platelet-derived growth factor
PF: pulmonary fibrosis
PFD: pirfenidone
PVDF: polyvinylidene difluoride filter
Q-PCR: quantitative PCR
RNA-Seq: RNA sequencing
TGF: transforming growth factor
TNF: tumor necrosis factor
